# The genetically engineered drug rhCNB induces apoptosis via a mitochondrial route in tumor cells

**DOI:** 10.18632/oncotarget.19507

**Published:** 2017-07-22

**Authors:** Yang Yang, Huan Yang, Jinju Yang, Li Li, Benqiong Xiang, Qun Wei

**Affiliations:** ^1^ Department of Biochemistry and Molecular Biology, Beijing Normal University, Beijing Key Laboratory, Beijing 100875, People’s Republic of China

**Keywords:** rhCNB, mitochondrial apoptosis, Bcl-2 family, tumor targeting, anti-tumor drug

## Abstract

The calcineurin B subunit (CNB) has antitumor activity. We showed previously that recombinant human CNB (rhCNB) also had strong anti-tumor activity *in vivo*, and was thus a promising candidate anti-tumor drug. It appeared to kill tumor cells via immunomodulation. Here, we show that rhCNB inhibits the proliferation of human hepatoma HepG-2 cells, resulting in their apoptosis. Exogenous CNB was found to localize to mitochondria in tumor cells and activate the mitochondrial apoptosis pathway, as indicated by a decrease of mitochondrial transmembrane potential, release of cytochrome C and activation of caspase-9, which then activates caspase-3. At the same time Bcl-2 &Bcl-xL expression decreased, Bim expression increased, and Bax was activated. Interaction between rhCNB and Bcl-xL was detected, which may inhibit the function of Bcl-xL. Long-term tumor targeting was also observed in nude mice. These data deepened our understanding of the anti-tumor mechanism of rhCNB and provided guidance for its drug development.

## INTRODUCTION

Calcineurin (CN) is a calcium- and calmodulin-dependent serine/threonine protein phosphatase [1, 2], whose function and structure have been widely studied. It plays a central role in activating immune cells and is the target of the much-used immunosuppressive agents cyclosporin A (CsA) and tacrolimus (FK506) [3, 4]. It is also involved in transcriptional and post-transcriptional regulation of apoptosis [5, 6]. CN is a heterodimer composed of A and B subunits. The A subunit (CnA) is a 61 kD catalytic subunit, with three regulatory domains: a CNB-binding domain (BBH), a CaM-binding domain (CBD) and an auto-inhibitory domain (AID). The B subunit (CNB) is a 19 KD regulatory subunit with four Ca^2+^ binding sites [7–9]. These regions and sites are essential for Ca^2+^ regulation of CN activity.

The basic role of CNB is to regulate the phosphatase activity of CN and stabilize its structure, allowing it to participate in cell metabolism and signal pathway regulation. However, CNB also has other, non-regulatory, roles. It is involved in apoptosis and the proteasome pathway by interacting with heat shock protein 60, tubulin, pro-caspase 3 and other proteins [10–11]. CNB-deficient mice have a high risk of squamous cell carcinoma, suggesting that CNB has anti-tumor activity [6]. We have shown that recombinant human CNB (rhCNB) has a strong anti-tumor effect in a variety of tumor models. It significantly extended the survival of mice bearing H_22_ hepatoma ascites and S180 sarcoma tumors [12], and it had a pronounced antitumor effect on the B16 lung metastasis model in C57 mice [13]. These results indicate that rhCNB holds promise as an anti-tumor drug, and rhCNB has been developed as a genetically-engineered drug, patented in China and the US [14, 15]. In terms of its anti-tumor mechanism, studies have shown that it activates the immune system by inducing the maturation and activation of dendritic cells and enhancing their antigen-presenting activity [16]; it also promotes the proliferation of peritoneal macrophages and stimulates their phagocytic activity synergistically with IFN-γ [13]; furthermore it interacts with integrin αM and increases TNF-related apoptosis-inducing ligand (TRAIL) expression in macrophages, thereby inducing tumor cell apoptosis [17, 18]. Studies have also shown that overexpression of CNB can significantly increase TNF-α/CHX-induced apoptosis, which may be achieved by affecting the function of mitochondria [19], and exogenous CNB rapidly enters cells through TLR4 receptors and induces the secretion of cytokines, but have certain cytotoxicity in some TLR4-rich tumor cells [20]. Interestingly, we observed that exogenous CNB rapidly enters tumor cells and becomes located in mitochondria. This observation led us to ask whether rhCNB can induce apoptosis of tumor cells directly, aside from any action on the immune system, and if so, what mechanism is involved.

There are two main pathways of apoptosis. The death receptor signaling pathway, also known as the extrinsic pathway, is activated by binding of specific ligands to death receptors on the cell membrane; this recruits the Fas-related death domain (FADD) and caspase-8 to form the death-inducing signaling complex (DISC), which then leads to activation of caspases and induces apoptosis [21, 22]. The mitochondrial apoptotic pathway, the so-called intrinsic pathway, is often initiated by the activation of pro-apoptotic members (eg Bax, Bak) on the OMM to form channels, allowing the escape of mitochondrial contents, and finally apoptosis is induced by a cascade of reactions [23–25]. The anti-apoptotic members (eg Bcl-2, Bcl-xL, Bcl-W) are generally sequestered within the OMM to inhibit activation of the pro-apoptotic ones [26, 27], while the BH3-only proteins (eg Bid, Bim, Bad) either interact with the anti-apoptotic proteins to inhibit their function, or activate pro-apoptotic proteins directly [28–32]. Thus the balance of Bcl-2 family members can decide cell fate.

In order to test the hypothesis that rhCNB can induce apoptosis of tumor cells directly, we treated HepG-2 cells with rhCNB and found that it inhibited their proliferation and activated mitochondrial apoptosis pathway. We investigated for the first time the cytotoxicity of rhCNB on hepatoma cells and the important inhibitory effect on Bcl-xL activity in this process, which further elucidated the anti-tumor mechanism of rhCNB and promoted the drug development process of rhCNB. Meanwhile, we also found that rhCNB has the property of tumor targeting *in vivo*, providing a new perspective for further research.

## RESULTS

### Exogenous CNB inhibits the proliferation of HepG-2 cells by apoptosis

**Figure 1:**
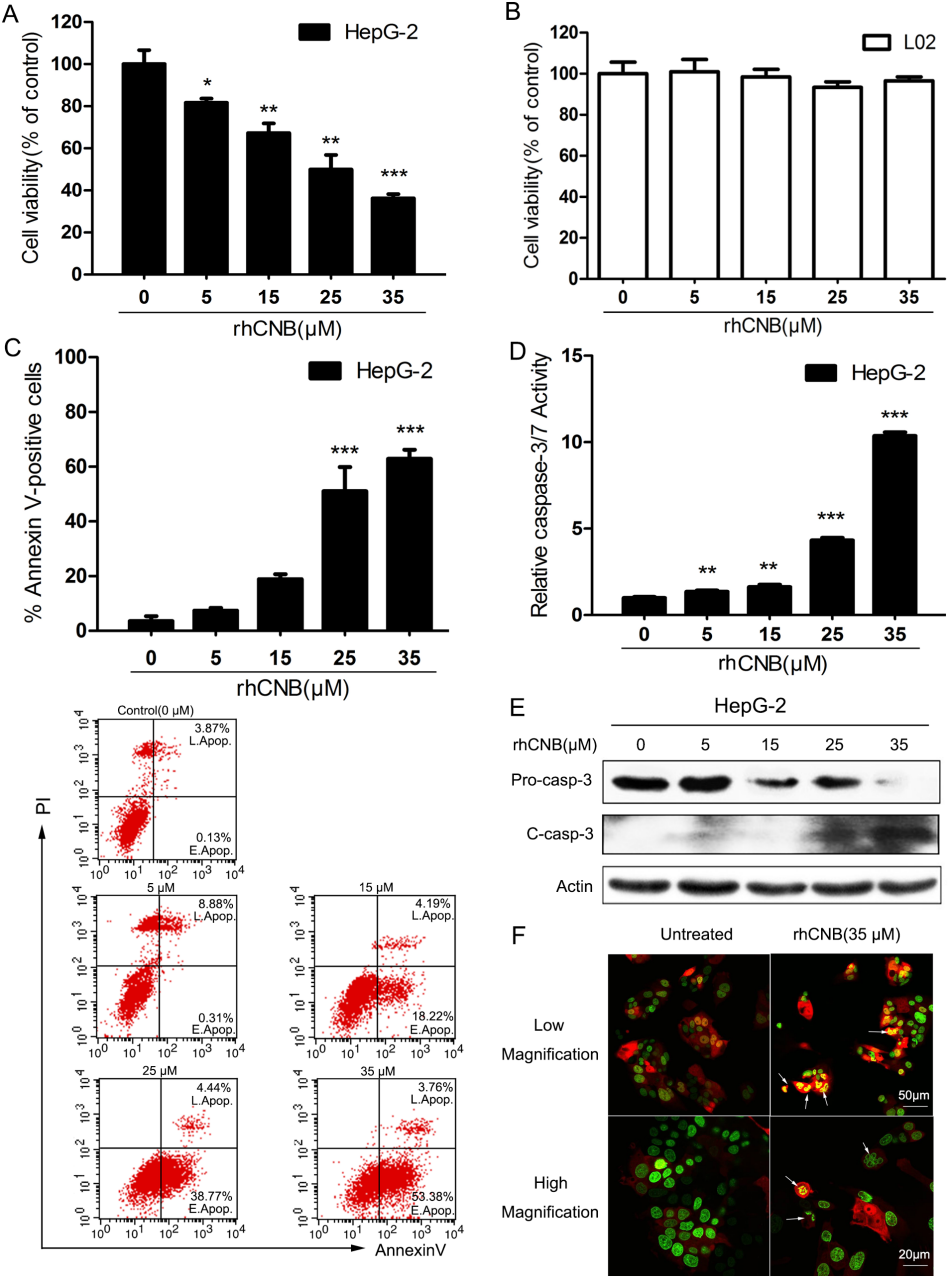
The antiproliferative activity of rhCNB is due to apoptosis **(A, B)** Dose-dependent decrease in cell viability. HepG-2(A) and LO2(B) cells were treated with increasing concentrations of rhCNB (0-35μM) for 48h. The relative cell viability was shown as mean ± SD, *P<0.05; **P<0.01; ***P<0.001 (compared with control). **(C)** rhCNB increases apoptosis in a dose-dependent manner. HepG-2 cells were treated with varying concentrations of rhCNB (0-35μM) for 48h before stained with annexin V assay. Detailed apoptosis analyses are shown in the histogram, representing the mean percentages ± SD of apoptosis in three independent experiments, ***P<0.001 (compared with control). The lower panel shows scatter plots of the percentages of early and late apoptosis in one experiment. **(D)** Caspase-3/7 activity is increased by rhCNB. Caspase-3/7 catalytic activity was analyzed in HepG-2 cells treated with varying concentrations of rhCNB. Shown are mean ± SD, **P<0.01; ***P<0.001 (compared with control). **(E)** Cleaved caspase-3 levels observed by Western blotting. **(F)** Condensation of chromatin material and fragmentation of nuclei in apoptotic cells. HepG-2 cells stably transfected with the nuclear marker Histone H2B-EGFP and cytoplasmic marker DsRed2 were treated with 35μM rhCNB for 48h and examined with a Zeiss LSM700 confocal laser scanning microscope.

To examine the antiproliferative activity of rhCNB, HepG-2 cells were exposed to various concentrations of rhCNB for 48h, after which the extent of cell death was monitored by CCK8 assay. The cytotoxicity of rhCNB for normal human LO2 liver cells was also evaluated. The relative viability for HepG-2 cells was 81.67±3.48% at 5μM and decreased to 34.23±3.44% at 35μM (Figure 1A), while little to no cytotoxicity was observed for LO2 cells (Figure 1B), indicating that rhCNB has low general toxicity, and this is not due to absence of incorporation as rhCNB can enter both HepG-2 and LO2 cells (Supplementary Figure 1). A dose-dependent increase in the percentage of apoptotic HepG-2 cells from 7.40 ± 1.35% to 62.86 ± 4.72% was observed after treatment with 5-35μM of rhCNB (Figure 1C) determined by Annexin V binding, suggesting that most of the antiproliferative activity of rhCNB is mediated by apoptosis. Meanwhile, activation of caspase-3, an important physiological and biochemical marker of apoptosis was detected. RhCNB increased the level of cleaved caspase-3 in a dose-dependent manner (Figure 1E) and resulted in a significant increase of caspase-3 activity (Figure 1D). Furthermore, morphological changes induced by rhCNB were observed in DsRed2/HistoneH2B-EGFP dual-labeled HepG-2 cells (Figure 1F): after incubation with 35μM rhCNB for 48 h, the nuclei of HepG-2 cells (green) were disrupted and overlapped with the cytoplasm (red) to produce a bright yellow color, indicating that apoptosis had occurred.

### Exogenous CNB enters tumor cells and localizes in mitochondria

**Figure 2:**
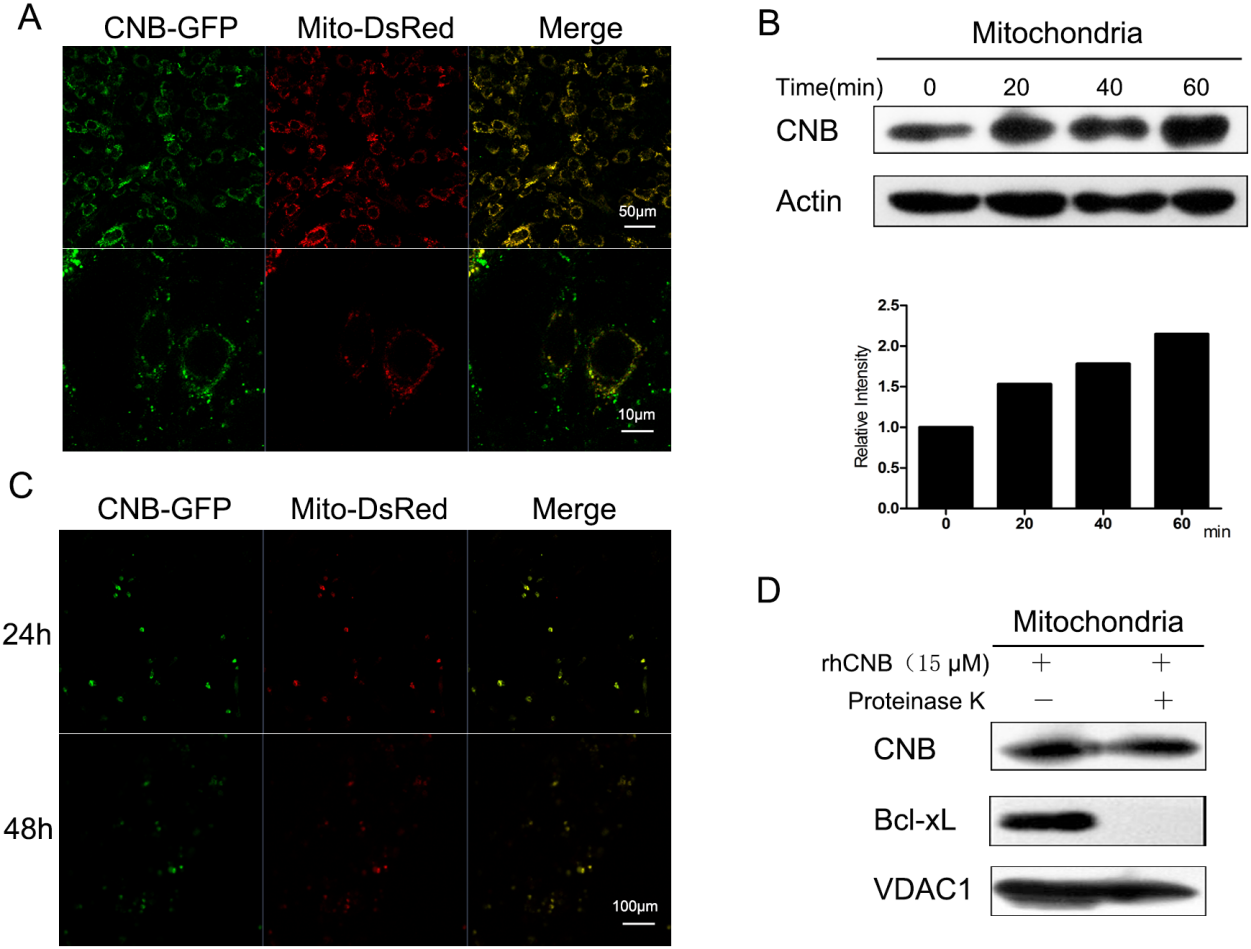
Exogenous CNB becomes localized in mitochondria (**A**) rhCNB co-localizes with mitochondria. Sk-hep-1 cells were transfected with the mitochondrial marker Mito-DsRed for 48h and treated with 5μM CNB-GFP for 30 min, then visualized using a Zeiss LSM700 confocal laser scanning microscope. The scale bars represent 50μm (upper) and 10μm (lower). **(B)** Mitochondrial rhCNB at different times. Sk-hep-1 cells were incubated with 10μM rhCNB for 0, 20, 40, 60 min after which mitochondria were isolated for Western blotting. The graph represents the mean of the densitometric intensities of the visualized bands. **(C)** The persistence of rhCNB in mitochondria. Sk-hep-1 cells were transfected with the mitochondrial marker Mito-DsRed for 48h and treated with 5μM CNB-GFP for 24h and 48h, then visualized using a Zeiss LSM700 confocal laser scanning microscope. The scale bar represents 100μm. **(D)** rhCNB can enter the inner space of mitochondria. Mitochondria isolated from HepG-2 cells treated with 15μM rhCNB for 6h were digested with proteinase K for 30 min, and the lysates were subjected to western blotting.

It has been shown that rhCNB rapidly enters tumor cells [20]. To see if it becomes associated with mitochondria we incubated Mito-DsRed-transfected sk-hep-1 cells with exogenous CNB-GFP. As shown in Figure 2A, CNB-GFP co-localized with Mito-DsRed, indicating that the exogenous rhCNB became localized to mitochondria. To confirm this, rhCNB was incubated with sk-hep-1 cells for 20, 40 or 60 min, and mitochondrial rhCNB content was measured. RhCNB became associated with the mitochondria; its level increased with time (Figure 2B) and remained co- localized with mitochondria for 24 and 48h (Figure 2C), suggesting that it may enter the inner space of mitochondria. We therefore digested the outer mitochondrial membrane protein with protease K. As shown in the Figure 2D, rhCNB was indeed still found in the mitochondria (upper panel) while the mitochondrial outer membrane protein Bcl-xL had been lost.

### Mitochondrial damage and caspase activation are involved in rhCNB-induced apoptosis

**Figure 3:**
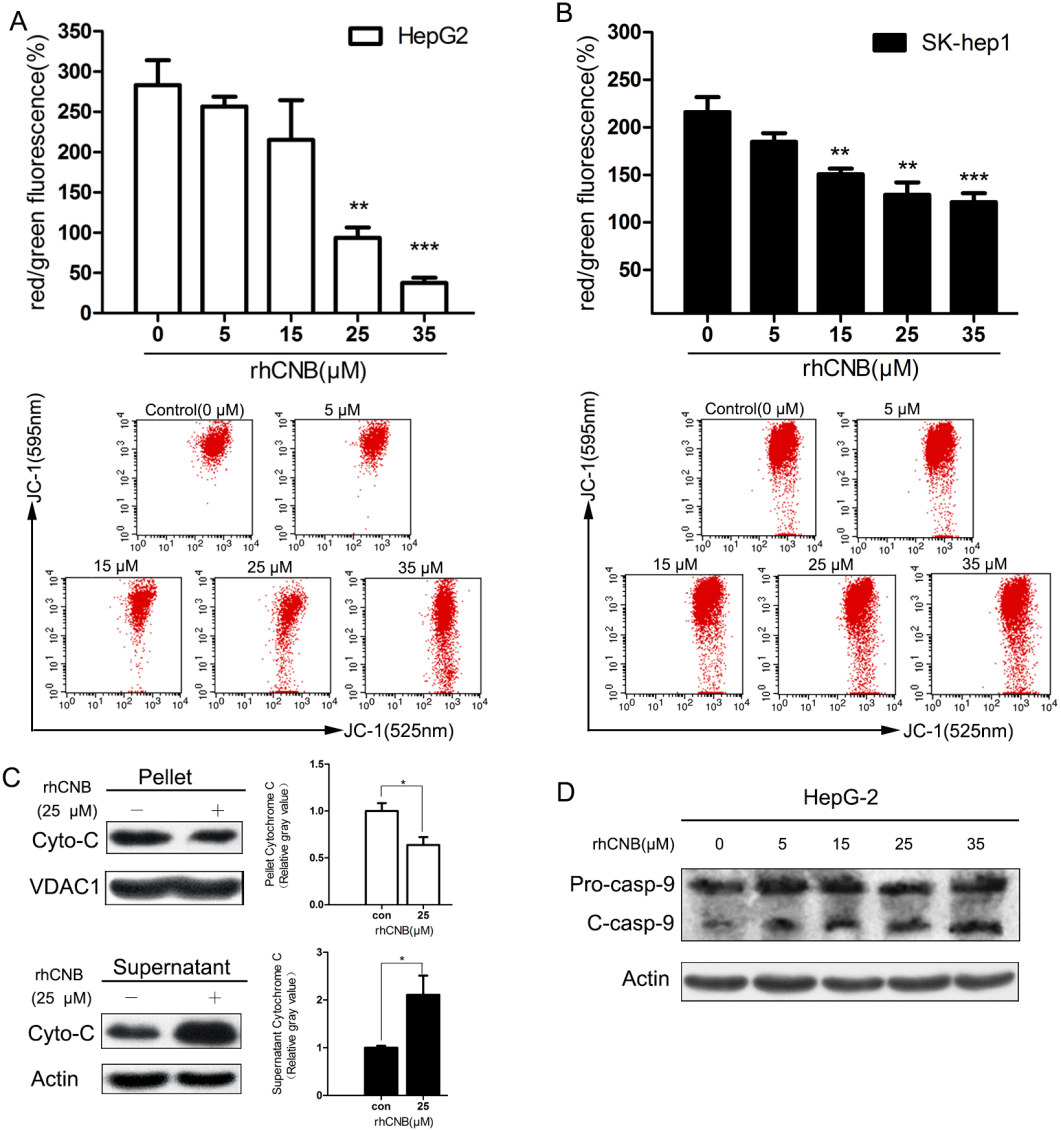
Exogenous CNB damages HepG-2 and Sk-hep-1 mitochondria HepG-2 cells **(A)** and SK-hep-1 cells **(B)** were treated with increasing concentrations of rhCNB (0-35μM) for 24h. Cells were harvested, stained with JC-1 dye and analyzed by flow cytometer. The mitochondrial membrane potential was represented by the ratio of red to green fluorescence intensity (100%), where red represents JC-1 aggregates in intact mitochondria and green represents green fluorescing monomers in the cytosol. Bar diagram shows the mean mitochondrial membrane potentials ± SDs of three independent experiments, **P<0.01; ***P<0.001 (compared with control). The lower panel illustrates the loss of red fluorescence with increasing rhCNB in one experiment. **(C)** rhCNB causes release of cytochrome C to the cytosol. HepG-2 cells were treated with 25μM rhCNB for 48h and cytochrome C release was assessed by Western blotting of pellet versus supernatant. Bar diagram shows the mean mitochondrial or cytosolic cytochrome C ± SDs of three independent experiments, *P<0.05. **(D)** Cleaved caspase-9 levels observed by Western blotting.

In response to rhCNB treatment, HepG-2 and Sk-hep-1 cells were found to undergo a dose-dependent increase in mitochondrial depolarization, reflected by a decrease in mitochondrial transmembrane potential (Figure 3A, 3B). HepG-2 cells were the more sensitive of the two cell types: their membrane potential was reduced by 72.6% when incubated with 35μM rhCNB. This was accompanied by profound mitochondrial damage, reflected in a marked increase in cytosolic cytochrome (Figure 3C). In addition, rhCNB was found to trigger activation of the initiator caspase, caspase-9, as demonstrated by a decrease in the pro-caspase form and the appearance of the corresponding cleaved form. These observations suggest that activation of the mitochondrial pathway is responsible for the induction of apoptosis by rhCNB.

### Exogenous CNB modulates the expression and function of Bcl-2 family members

**Figure 4:**
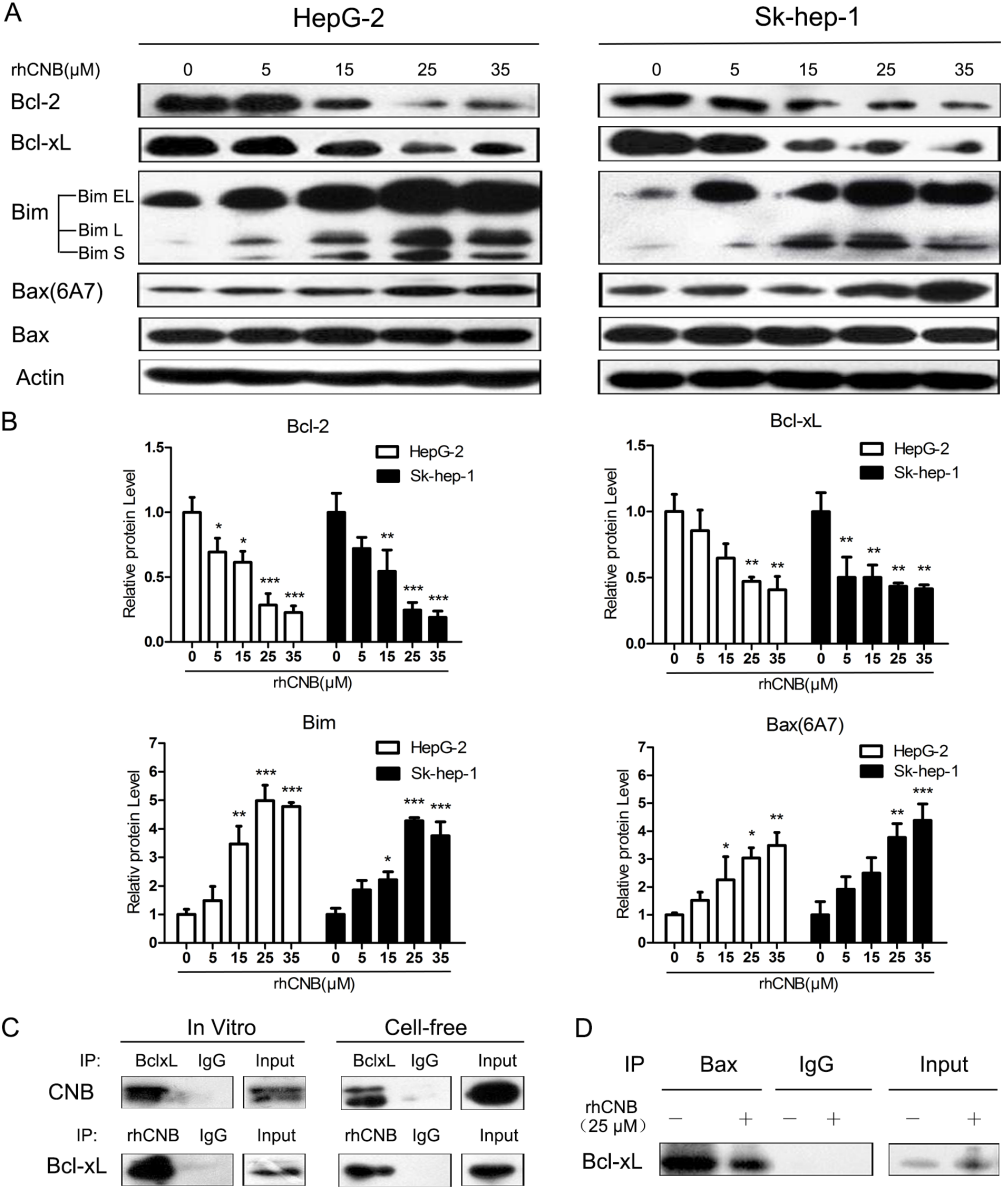
rhCNB alters the expression of Bcl-2 family members and weakens the interaction of Bcl-xL and Bax **(A)** HepG-2 cells were exposed to rhCNB for 48h after which lysates were prepared and subjected to Western blot analysis using the designated Abs. **(B)** Densitometry of Bcl-2, Bcl-xL, Bim, Bax(6A7) Western blots (relative to untreated control), in HepG-2 and Sk-hep-1 cells treated with different concentrations of rhCNB for 48h. Values represent mean relative band densities ± SDs of three independent experiments, *P<0.05; **P<0.01; ***P<0.001 (compared with control). **(C)** rhCNB and Bcl-xL interact *in vivo* and *in vitro*. HepG-2 cells treated with 15μM rhCNB for 6h were lysed and immunoprecipitated with Abs against Bcl-xL and CNB (left panel). Purified recombinant Bcl-xL and rhCNB proteins were incubated for 30 min and immunoprecipitated with Abs against Bcl-xL and CNB (right panel). The immunoprecipitates were separated by SDS-PAGE and immunoblotted with CNB and Bcl-xL Abs. **(D)** HepG-2 cells were treated with 25μM rhCNB for 6h after which they were lysed and immunoprecipitated with Ab against Bax, and the immunoprecipitates were separated by SDS-PAGE and immunoblotted with Bcl-xL Abs.

Members of the Bcl-2 family are thought to regulate mitochondrial outer membrane integrity, thus controlling apoptosis. We therefore investigated whether exogenous CNB influences the expression and function of Bcl-2 family members. It was found that rhCNB dose-dependently decreased the protein levels of Bcl-2 and Bcl-xL, both being anti-apoptotic proteins (Figure 4A, 4B). Meanwhile, rhCNB also increased the protein level of Bim, a BH3-only protein (Figure 4A, 4B) that binds to various Bcl-2 anti-apoptotic proteins and inhibits their function [33–34]. There were three spliced forms of Bim (BimEL, BimL, BimS) in HepG-2 cells, but only the first two in SK-hep-1 cells, which is generally the case [35]. Results of real-time quantitative PCR showed consistent trends in mRNA levels (Supplementary Figure 2), indicating that the changes in the protein levels may be partly due to effects on gene expression. When the balance of anti- and pro-apoptotic proteins tilts towards the latter, apoptosis is induced. In view of the important role of the multi-domain protein Bax in apoptosis [36], we also examined its activation in HepG-2 and Sk-hep-1 cells and found that rhCNB promoted formation of the active Bax conformation (6A7), with no major change in the level of the protein (Figure 4A). We may therefore suppose that activation of Bax by rhCNB is mediated by down-regulation of Bcl-2 and Bcl-xL and up-regulation of Bim, which further activates the mitochondrial apoptotic pathway.

However, the role of rhCNB localization in the mitochondria remains unclear. Is it possible that it interacts with proteins in the mitochondria, which in turn affect mitochondrial function? This idea led us to test whether rhCNB interacted with Bcl-2 or Bcl-xL, two anti-apoptotic Bcl-2 proteins located on the outer membrane of mitochondria. Co-immunoprecipitation experiments showed that rhCNB failed to bind to Bcl-2 (Supplementary Figure 3), but it did bind to Bcl-xL, *in vitro* as well as in cell-free system (Figure 4C). As Bcl-xL has been reported to inhibit the translocation of pro-apoptotic Bax from the cytoplasm to mitochondria [27], we conjectured that binding of rhCNB to Bcl-xL might disrupt the interaction between Bcl-xL and Bax. As shown in Figure 4D, binding of Bcl-xL to Bax in HepG-2 cells was indeed weakened in response to rhCNB, and this may promote activation of Bax.

In summary, rhCNB disrupts the stability of mitochondria by altering the expression and functioning of members of the Bcl-2 family, so leading to apoptosis.

### Exogenous CNB targets tumors

**Figure 5:**
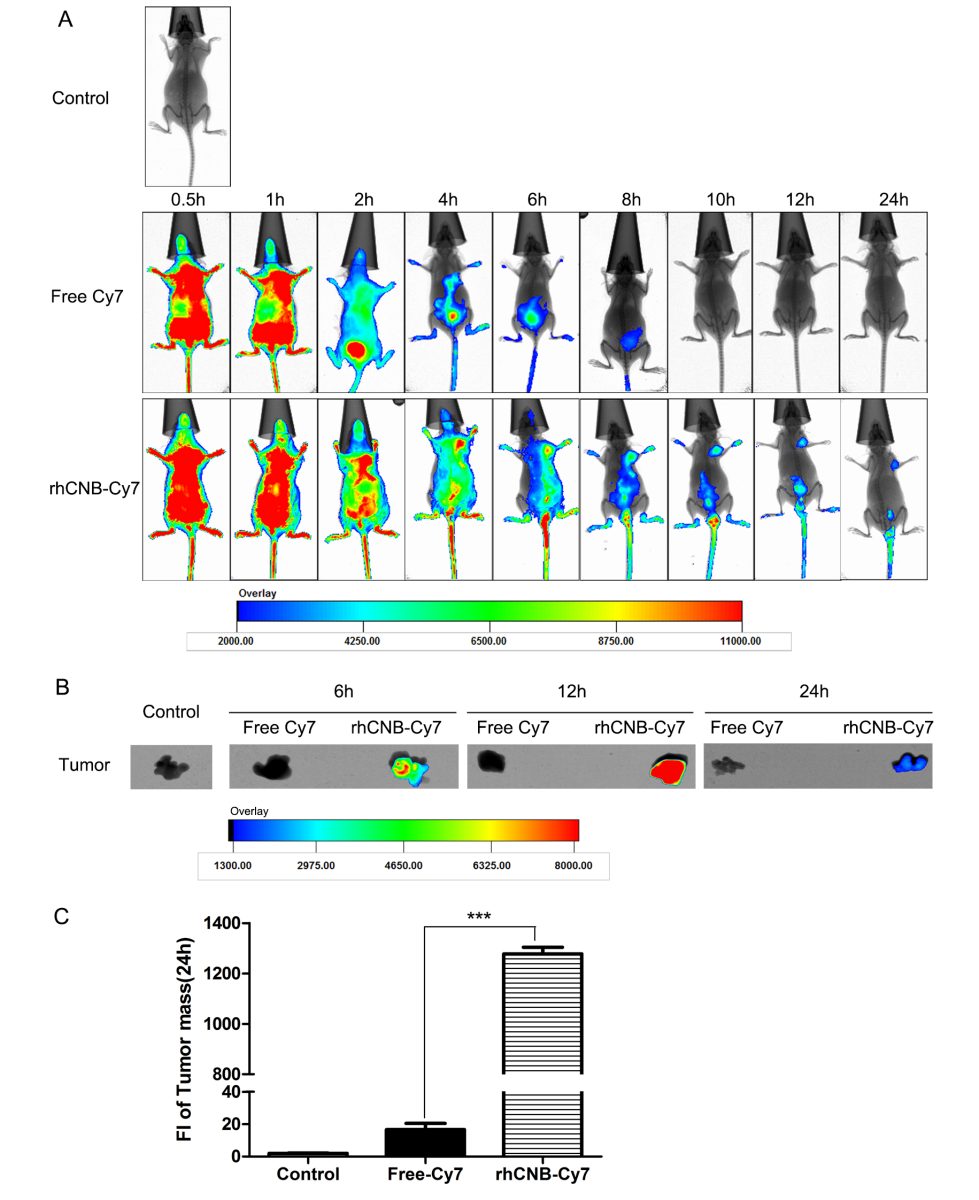
rhCNB localizes to tumors *in vivo* **(A)** *In vivo* real-time imaging of the tumors arising from HepG-2 cells in nude mice after intravenous injection of free Cy7 or rhCNB-Cy7. **(B)** *Ex vivo* optical images of HepG-2 tumor masses from mice at 6h, 12h, and 24h. Note: Cy7 refers to cyanine 7, which is used as a fluorescent probe. **(C)** Quantitative analysis of fluorescence intensity in tumor masses. Bar diagram represents the mean fluorescence intensity ± SDs, ***P<0.001.

Distribution of drugs *in vivo* is often associated with their pharmacological effects, so we would like to study the distribution of rhCNB in mice, especially at the tumor site. Then *in vivo* imaging observations were made on tumors formed by HepG-2 cells. We injected fluorescent dye Cy7-labeled rhCNB or free Cy7 into tumor-bearing nude mice by intravenous injection, and observed the distribution of fluorescence in mice at different times. The *in vivo* imaging results demonstrated that once injected, Cy7 and rhCNB-Cy7 rapidly spread throughout the mice via the bloodstream. Cy7 was quickly discharged via metabolism, after brief retention in the bladder and other metabolic organs, while Cy7-labeled rhCNB was clearly localized in the tumor tissue 6 h after injection and persisted up to 24 h (Figure 5A). *Ex vivo*-fluorescent images and statistical analysis of the excised tumors confirmed the retention of Cy7-labeled rhCNB in the tumor masses (Figure 5B, 5C). These results indicate that rhCNB localizes to tumor masses *in vivo*, and this long-term localization may contribute to the safety of rhCNB.

## DISCUSSION

Large-scale production of the antitumor candidate drug rhCNB has been initiated. The product is of high purity and stability, and its molecular weight, peptide map, isoelectric point and other indicators demonstrate that it is identical to native CNB [14, 15]. In order to accelerate the development of rhCNB, we explored the mechanism of its anti-tumor effect in this study. Previous studies have indicated that rhCNB activates the immune system, and thus exerts an anti-tumor effect [13, 16–20]. However, we now provide evidence that it can kill human hepatoma cells directly via a mitochondria dependent way.

We have shown that apoptosis seems to be the primary mechanism of rhCNB-induced cell death, as HepG-2 cells showed obvious morphological damage like nuclei fragmentation, associated with early and late apoptotic events. rhCNB caused depolarization of mitochondria in a dose dependent manner, with a profound cytochrome C release and cleavage of initiator caspases-9, a characteristic for numerous stimuli that cause apoptosis via the intrinsic pathway involving mitochondria [23–24]. Then, activation of the terminal caspase-3, one of the key effector molecules in apoptosis was also observed. These results preliminarily clarify the actions of rhCNB in apoptosis induction.

Since the activity of Bcl-2 family members can be affected or modulated by many antineoplastic therapies [37–40], we examined the contribution of the major members in this family to rhCNB-induced apoptosis. In a variety of tumor cells, Bcl-2 and Bcl-xL are highly expressed to evade apoptosis. Studies have shown that up-regulation of BCL-xL increases the chemo-resistance of tumor cells [41–43] and expression of Bcl-2 always correlates with sensitivity to anticancer drugs [44–45]. Inhibition of Bcl-2 and/or Bcl-xL has been shown to induce spontaneous apoptosis of tumor cells [46–48]. In this study, we found that rhCNB treatment resulted in a substantial decrease in expression of Bcl-2 and Bcl-xL in hepatoma cells, while no obvious reduction in normal liver cells (Supplementary Figure 4B). This is explainable as some tumor cells have a higher mitochondrial priming state than normal cells, thus are more sensitive to chemotherapy [49, 50]. Simultaneously, rhCNB treatment led to an increase in the ratio of Bax/Bcl-2, characteristic of activation of the mitochondrial apoptotic pathway [47], the *active* form of Bax did increase; this may have been due at least in part to the down-regulation of Bcl-2 and Bcl-xL, as they are thought to inhibit the aggregation of Bax on the OMM [27, 46]. We also detected up-regulation of Bim in response to rhCNB, an activator protein that inhibit anti-apoptotic Bcl-2 proteins as well as directly activating Bax and Bak [30, 31, 51], with Bax preference [52]. Once activated, Bax forms channels in the OMM, leading to mitochondrial outermembrane permeabilization (MOMP) to initiate mitochondrial apoptosis [23, 24]. As stated before, our data support this mechanism for the effect of rhCNB. Thus we hypothesize that rhCNB reduces the expression of Bcl-2 and Bcl-xL so allowing Bax to accumulate in the OMM, and they also upregulate Bim to further activate Bax, which then triggers the mitochondria apoptosis pathway.

We showed that rhCNB becomes located in mitochondria once it enters hepatoma cells. Mitochondria play an important role in cell metabolism, apoptosis, calcium regulation and other physiological activities [53], and the presence of rhCNB may affect their function. Our results show that rhCNB binds to the mitochondrial outer membrane protein Bcl-xL *in vitro* and in cell-free system. This may disrupt the interaction of Bcl-xL and Bax, interfering with inhibition of Bax by Bcl-xL and leading cells to be more sensitive to apoptosis. It seems that rhCNB can also enter the inner mitochondria space, but the role it plays there is unknown. It has been shown to bind to isolated mitochondria in a Ca^2+^-dependent manner [19] and cause endoplasmic reticulum stress (unpublished data). As a result, when it enters mitochondria, it will presumably cause mitochondrial calcium overload and influence the opening of the mitochondrial permeability transition pore (PTP) [54]. Future studies of the role of Ca^2+^ in rhCNB-induced apoptosis should lead to a better understanding of the direct killing mechanism of rhCNB.

As a newly reported candidate drug for tumor treatment, rhCNB not only has the excellent ability of tumor suppression, but also has the property of low toxicity *in vitro* and *in vivo*. Previous acute toxicity experiments indicated that mice can endure at least 50-fold the physiological dose of rhCNB [12]. LD50 of acute toxicity test on cynomolgus monkey is 1600 mg/kg, 300-fold of the normal dose. Long-term toxic experiment showed that rhCNB has no significant toxicity on immune organs and other vital organs of rats and cynomolgus monkeys (completed by a qualified center for drug safety evaluation and research). In this study, we confirmed it again in LO2 cells (Figure 1B) and mouse primary hepatocytes (Supplementary Figure 4A). We also showed that rhCNB can persist in the tumor masses of nude mice for as long as 24 hours, which may contribute to the safety of rhCNB *in vivo*. This is another significant advantage of rhCNB as the utility of many antitumor drugs was limited by lack of specificity, and researchers have conjugated some drugs with carriers to target their delivery [55–57]. In addition, it could be useful to explore which parts of the rhCNB structure lead to its tumor-targeting. RhCNB might also be conjugated with other drugs to enhance their anti-tumor effect and reduce side effect.

We have reported for the first time that rhCNB can activate the mitochondrial apoptosis pathway by modulating the expression and activity of members of the Bcl-2 family, and induce apoptosis in human hepatoma cells. It also appears to be specifically taken up by HepG-2 solid tumors. Our findings have deepened our understanding of the anti-tumor mechanism of rhCNB and provided guidance for its development and application.

## MATERIALS AND METHODS

### Materials

Recombinant human CNB [EU <0.25U] was provided by Haikou Qili Pharmaceutical Co., Ltd. The anti-caspase-3(8G10), anti-cleaved caspase-3(Asp175), anti-caspase-9(9502), cytochrome C (136F3), Bcl-2(50E3), Bcl-xL(54H6) and Bim(C34C5) antibodies were purchased from Cell Signaling Technology; the anti-Bax(610982) and anti-Bax(6A7)(556467) antibodies were purchased from BD Biosciences; the anti-CNB antibody was prepared by our laboratory [58]. The Ni-NTA columns were from Nano-Micro Co, Ltd. (C05W206E). The following reagents were also used: lipofectamine 2000 (Life Technologies; 11668-019), a caspase-3/7 Live Cell Fluorometric Assay Kit (KeyGEN BioTECH; KGAS037-50), and Cy7 (Fanbo Biochemicals; 943298-08-6).

### Cells and cell culture

The following cells lines were used: HepG-2, Sk-hep-1 (ATCC), LO2(kindly donated by Professor Zhou Gangqiao, Academy of Military Medical Sciences), DsRed2/HistoneH2B-EGFP labeled HepG-2 cell (Anti-Cancer Biotech (Beijing)). LO2 were cultured in DMEM (Gibco; C11965500BT), other cell lines were cultured in MEM (Gibco; C11095500BT), containing 10% fetal bovine serum (Gibco; 10099-141). All cells were maintained at 37°C in a humidified incubator with 5% CO_2_.

### CCK-8 assay

Briefly, 8×10^3^ of HepG-2 cells or 1×10^4^ LO2 cells were seeded in wells of a 96-well plate, with medium as a blank-control. After 24 h incubation, cells were exposed to increasing concentrations of CNB (0–35μM) for an additional 48h. Then added 10μL of CCK8 working solution per well (including Blank) for 1.5-2h incubation in the dark, and measured the absorbance at 450nm. The relative cell viability (%) was calculated as the following formula: [A (drug treated group) -A (blank)] / [A (untreated group) -A (blank)] ×100 (wherein A represents absorbance at 450nm). There was a minimum of four sets of reaction per group, and the results are presented as bar diagrams along with standard deviations (SDs).

### Caspase-3/7 activity

The activation of caspase-3/7 was quantified with a Caspase-3/7 Live Cell Fluorometric Assay Kit (KeyGEN BioTECH; KGAS037-50) in accordance with the manufacturer’s protocol. Cells were seeded in 12-well microtiter plates and treated with 0-35μM rhCNB for 48h, followed by substrate in Caspase 3/7 assay buffer for 45 min. Caspase-3/7 activities were calculated from the fluorescence measured in a Multi-Microplate Reader( BMG LABTECH).

### Analysis of apoptosis by flow cytometry

The percentage of cells undergoing apoptosis after treatment with rhCNB was determined using an Annexin V-FITC Apoptosis Detection Kit I (BD Bioscience; 556547). The kit uses FITC-conjugated Annexin-V to detect phosphatidylserine (PS) on the external membrane of apoptotic cells, and PI (propidium iodide) as a dead cells marker. HepG-2 cells (4×10^5^) were seeded in wells of a 6-well plate and incubated for 24 h. Following rhCNB treatment (0-35 μM, 48h), cells were harvested, washed with ice cold PBS and incubated with Annexin V-FITC and PI for 15 min at room temperature in the dark, and analyzed by flow cytometry.

### Measurement of mitochondrial transmembrane potential by flow cytometry

Mitochondrial membrane potential (ΔΨm) was measured with a JC-1 Apoptosis Detection Kit (KeyGEN BioTECH; KGA601). Briefly, 4×10^5^ HepG-2 cells and 2.5×10^5^ Sk-hep-1 cells were seeded in wells of a 6-well plate and incubated for 24 h. Following rhCNB treatment (0-35 μM, 24 h), they were harvested, washed with ice- cold PBS and incubated with JC-1 dye for 20 min at 37 °C in the dark. Cells were washed and samples were analyzed by flow cytometer after applying appropriate gates. The mean intensities of red and green fluorescence were recorded, and the ratio was considered the transmembrane potential (ΔψM).

### CNB-GFP fusion protein

CNB cDNA and a sequence coding for a linker (GGGSGGGS) were inserted into pet25b (+) to construct a recombinant plasmid carrying a GFP tag. The plasmid was transformed into *E. coli* BL21 (DE3), and the cells were induced with 1 mM IPTG (C9H18N5S, Merck) to initiate expression of the fusion protein. The cells were harvested and sonicated, and the protein was purified with Ni-NTA resin (C05W206E, Nano-Micro) and analyzed by SDS-PAGE.

### Co-localization analysis using confocal laser scanning microscopy

Confocal microscopy was performed with a Zeiss LSM700 laser scanning confocal microscope. HepG-2 or SK-hep-1 cells were seeded in 35-mm glass-bottom dishes (*In Vitro* Scientific; D35-20-1-N) and transfected with the indicated plasmids for 48 h. 5 μM CNB-GFP was then added to the cells and incubated for 30 min, followed by three washes with PBS. The distribution of fluorescence was determined.

### Western blotting and co-immunoprecipitation

For Western blotting, approximately 1× 10^6^HepG-2 or SK-HEP-1 cells were lysed with RIPA buffer. Clarified lysates were separated by SDS-PAGE, transferred to polyvinylidene fluoride membranes and incubated with the indicated antibodies (1.5h at room temperature or overnight at 4 °C). Proteins of interest were detected with secondary antibodies conjugated to HRP and Enlight™ Western Blot Detection Reagents (Engreen Biosystem; 29100). Pre-stained protein molecular weight markers (Thermo Scinentific; 26617) were included in each gel. To confirm equal loading of proteins in the gels, the blots were also probed with an antibody against β-actin (Applygen Technologies; C1313). For co-immunoprecipitation, 1 × 10^7^ HepG-2 cells were treated with rhCNB for 4h. Cells were lysed in ice-cold lysis buffer containing 1% NP-40, 50 mM Tris-HCl (pH 8.0), 150 mM NaCl and protease inhibitors (Roche; 4693116001) for 30 min. After centrifugation at 13,000 *g* for 30 min at 4 °C, lysates were pre-cleared by incubating (4°C, 2h) with 30 μL protein A/G Sepharose beads (GE Healthcare; 17-1279-01/17-0618-01). The precleared lysates were incubated overnight with 2-5 μg of the indicated antibodies, and immunocomplexes were captured with 40 μL protein A/G Sepharose beads. The presence of given proteins in these complexes was determined by Western blot analysis. Most primary antibodies were diluted to 1: 1000, while anti-cleaved caspase-3, anti-cleaved caspase-9 and anti-Bax(6A7) antibodies were diluted to 1:500. All secondary antibodies were diluted to 1: 5000.

### Detection of mitochondrial rhCNB

Mitochondrial and cytosolic fractions of HepG-2 or Sk-hep-1 cells incubated with rhCNB for the indicated times were prepared using a Cell Mitochondria Isolation Kit (Beyotime; C3601). The presence of rhCNB in mitochondria was determined by Western blotting. To test whether rhCNB enters the inner space of mitochondria, mitochondria were digested with digestion buffer (20 mM HEPES-KoH (PH7.4), 80 mM KOAc, 5 mM MgOAc, 250 mM sucrose and 20μM Proteinase K (Amresco; 0706)) on ice for 30 min and analyzed by Western blotting.

### Analysis of Cyto-C distribution by Western blotting

Mitochondrial and cytosolic fractions of HepG-2 cells incubated with rhCNB for the indicated times were prepared with a Cell Mitochondria Isolation Kit (Beyotime; C3601). The distribution of rhCNB was determined by Western blotting. VDAC1 was used as a mitochondrial protein reference, and actin as a cytoplasmic protein reference.

### *In vivo* imaging of rhCNB in HepG-2 tumors

Six-week-old female BALB/c nude mice were from Beijing Vital River Laboratory Animal Technology. All animal experimental procedures were conducted according to protocols approved by Ethic and Animal Welfare Committee (NO.CLS-EAW-2013-015) and were conducted in strict accordance with institutional guidelines. For tumor implantation, a total of 2×10^6^ HepG-2 cells were injected subcutaneously into the right flank of the mice. After the tumors reached approximately 800 mm^3^, the mice received free Cy7 or Cy7- labeled rhCNBvia the tail vein, and were scanned 0.5, 1, 2, 4, 6, 8, 10, 12 and 24 h later using a Kodak multimode imaging system (Carestream Health, Inc., USA). Mice were killed at 6, 12, 24 h, and the tumor masses were excised for detection of fluorescence signal intensity.

### Statistical analysis

Data are expressed as means± SDs. Statistical analysis was performed by one way ANOVA and Student’s t-test using SPSS 13.0 software. Values were considered significant at *P*≤ 0.05.

## SUPPLEMENTARY MATERIALS FIGURES


