# Supplementary material for: The genetically engineered drug rhCNB induces apoptosis via a mitochondrial route in tumor cells
Source: Oncotarget. 2017 Jul 22;8(39):65876–88. doi: 10.18632/oncotarget.19507 (PMC5630379; doi:10.18632/oncotarget.19507)
Supplement: Supplementary file 1 [file oncotarget-08-65876-s001.pdf]

The genetically engineered drug rhCNB induces apoptosis via a mitochondrial route in tumor cells

SUPPLEMENTARY MATERIALS

As seen in Supplementary Figure 1, rhCNB can quickly enter both HepG-2 and LO2 cells *in vitro*, which indicate the null effect on normal cells is not due to absence of incorporation.

The effect of rhCNB on the mRNA levels of 12 members of the Bcl-2 family were examined, and found that 3 of them underwent significant changes, namely antiapoptotic proteins Bcl-2, Bcl-xL and the pro-apoptotic BH3-only protein Bim (Supplementary Figure 2), while others (eg Bid) underwent no significant change (data not shown).

Supplementary Figure 3A shows that although CO-IP conditions were optimized, the non-specific binding was always strong (IgG group), suggesting that Bcl-2 protein may bind nonspecifically to the beads. Therefore we used microscale thermophoresis detection (MST) to measure any binding. However, rhCNB failed to interact with Bcl-2 (Supplementary Figure 3B).

As seen in Supplementary Figure 4, rhCNB has no or little toxicity on mice primary hepatocytes and no obvious reduction in Bcl-2 and Bcl-xL was detected.

SUPPLEMENTARY METHODS

RT-PCR and real-time qPCR analysis

Total RNA was extracted cells using Trizol reagent (Life Technologies; 15596-026. RT-PCR was performed by the two-step method. Quantitative PCR was performed using SYBR Green (Takara; RR420A). The primers used in this study were designed and synthesized by Invitrogen.

MicroScale thermophoresis detection of binding between Bcl-2 and rhCNB

The GFP-tagged Bcl-2 construct was transfected into 293T for 48h and lysed in IP lysis buffer for 30 min and the supernatant was cleared by centrifugation. The lysate was diluted depending on its fluorescence intensity. RhCNB was diluted with IP lysis buffer to a series of 16 1:1 dilutions (0.015-500μM), and GFP-tagged Bcl-2 from the lysate was added to each dilution and mixed. After 10-min, each mixture was added to Standard Treated Capillaries (NanoTemper Technologies). Termophoresis was measured using a Monolith NT.115 instrument (NanoTemper Technologies) at an ambient temperature of 25 °C with 5 s/30 s/5 s laser off/on/off times, respectively. The instrument parameters were adjusted to 50% LED power and 20% MST power. The data from three independently pipetted measurements were analyzed (NT.Analysis software version 1.5.41, NanoTemper Technologies) using the signal from Termophoresis+ T-Jump.

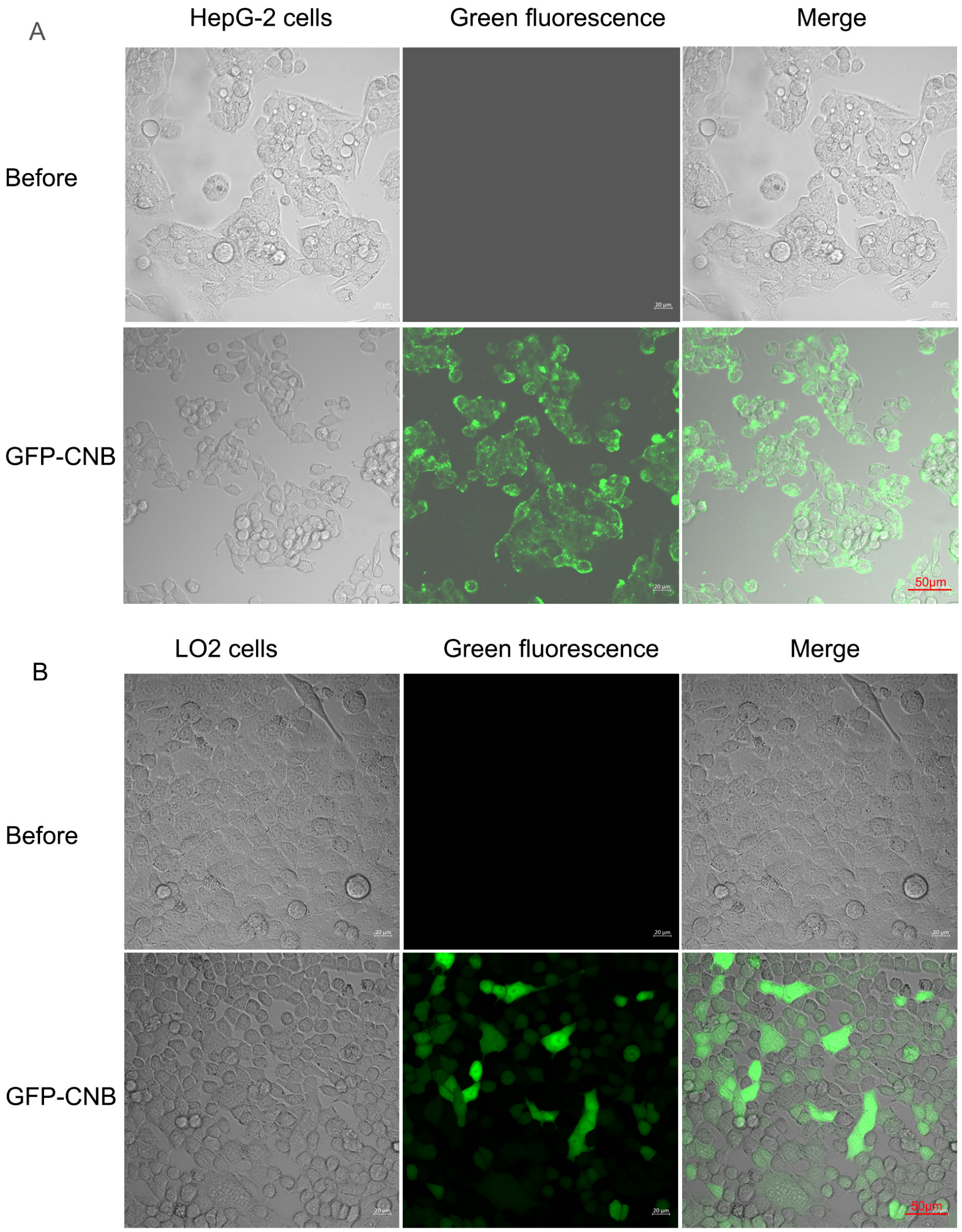

Supplementary Figure 1: Incorporation of rhCNB by HepG-2 and LO2 cells. HepG-2 cells **(A)** and LO2 **(B)** cells were incubated with 5μM CNB-GFP for 5 min, and then visualized using a Zeiss LSM700 confocal laser scanning microscope. The scale bar represents 50μm.

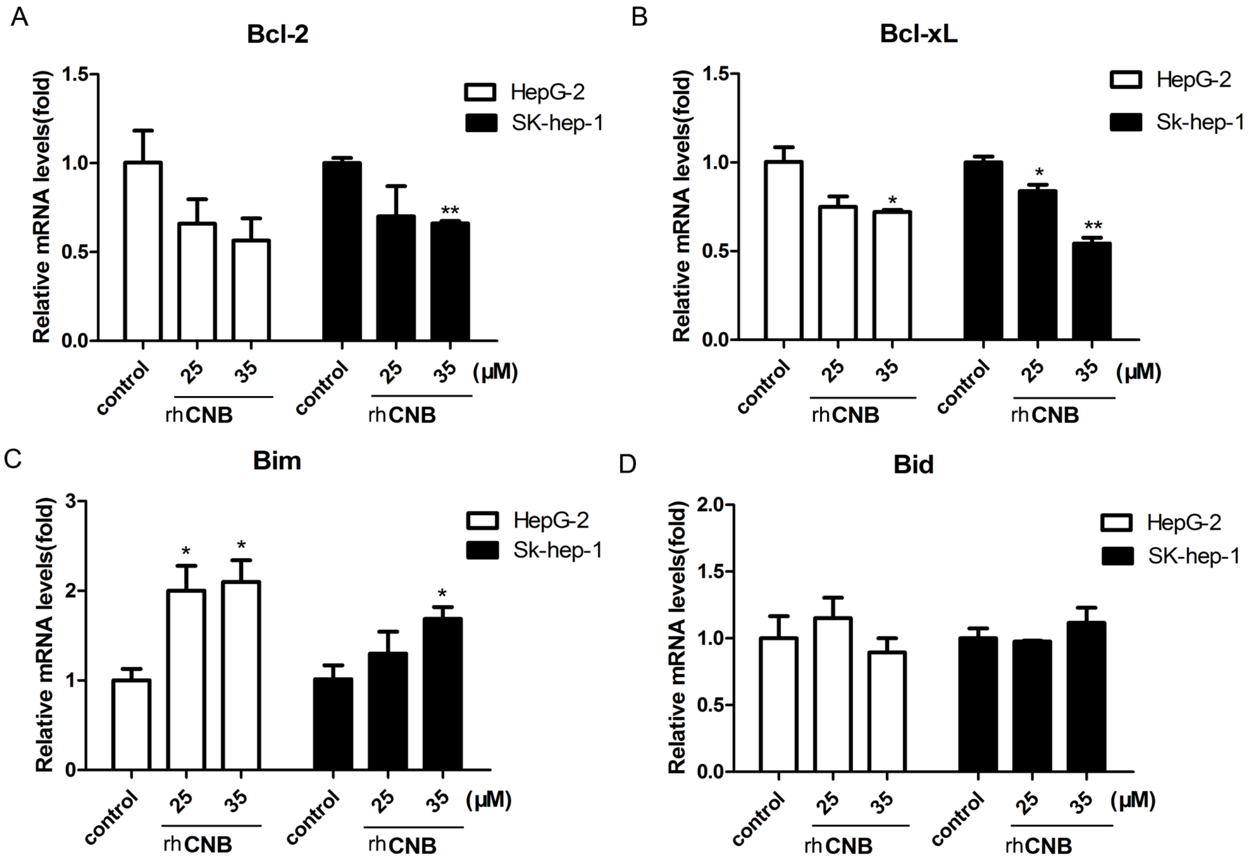

Supplementary Figure 2: Bcl-2 family mRNA levels in response to rhCNB stimulation. **(A-C)**: Real-time PCR of Bcl-2 and Bcl-xL(12h) and Bim(8h) in HepG-2 cells and SK-hep-1 cells incubated with rhCNB for the indicated times, and presented as fold increases relative to β-actin.

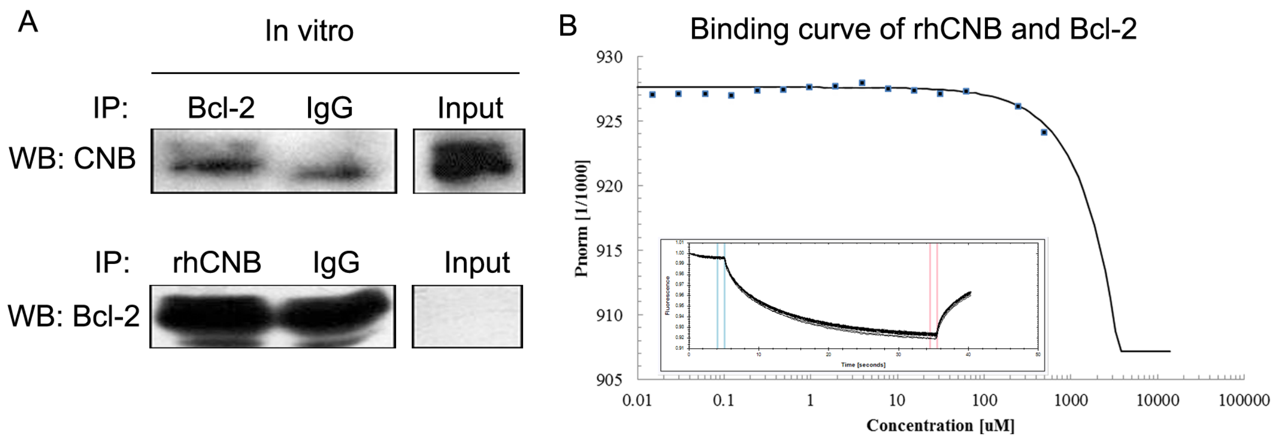

Supplementary Figure 3: Interaction of rhCNB with Bcl-2. (A) HepG-2 cells were treated with 15μM rhCNB for 6 h after which they were lysed and subjected to immunoprecipitation using Bcl-2 or CNB Abs, and the immunoprecipitates were analyzed by western blot. **(B)** rhCNB binding to Bcl-2 in lysates of transfected 293t cells assessed by MST analysis (see Methods).

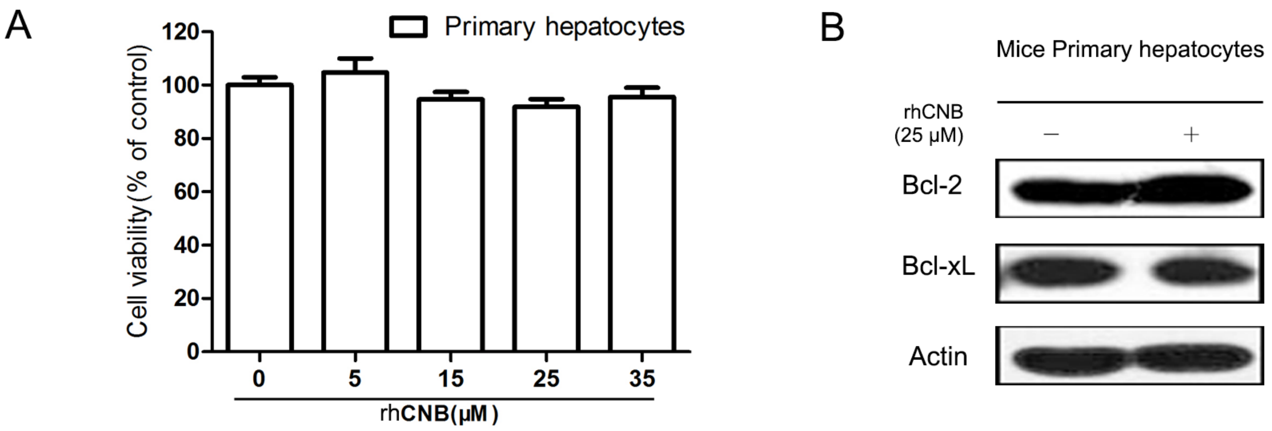

Supplementary Figure 4: The effect of rhCNB on primary hepatocytes. (A) Primary hepatocytes cells of ICR mice were treated with increasing concentrations of rhCNB (0-35μM) for 48 h. The percentage of growth inhibition rate was expressed relative to untreated control. **(B)** Primary hepatocytes cells of ICR mice were treated with 25μM rhCNB for 48 h after which cells were lysed and subjected to Western blotting.
